# The impact of treatment decisions on the diagnosis of bipolar disorders

**DOI:** 10.1186/2194-7511-2-3

**Published:** 2014-03-18

**Authors:** Emanuel Severus, Michael Bauer

**Affiliations:** Department of Psychiatry and Psychotherapy, TU Dresden, Dresden, 01307 Germany

## Editorial

The fifth edition of the Diagnostic and Statistical Manual of Mental Disorders (DSM-5) has broadened the criteria for specified bipolar disorders with the introduction of the diagnostic category of ‘other specified bipolar and related disorders’ (DSM-5: 296.89). In a prior editorial, we critically discussed how the way we diagnose bipolar disorders may impact treatment decisions, especially for patients with newly specified subthreshold bipolar II disorder (full depressive episode plus a hypomanic episode that does not meet the duration or number of symptoms required for a full episode) (Frances and Jones 2012; Phillips and Kupfer 2013; Severus and Bauer 2013; Zimmerman 2012; Angst 2013). In this editorial, we will examine how treatment decisions may, in turn, impact the diagnosis of bipolar disorders. Specifically, we will focus on how acute and prophylactic treatment of subthreshold bipolar II disorder may impact the rate of conversion to a diagnosis of bipolar I disorder.

In the DSM-5, subtypes of the nonorganic bipolar disorders are differentiated primarily by the number, duration, and intensity of the manic symptoms, as summarized in Table 1. To illustrate how the treatment of subthreshold bipolar II disorder may impact diagnosis, we will consider the case of a young patient with a history of a major depressive episode who visits his psychiatrist. Starting abruptly over the last 2 days, the patient felt increasingly ‘hyper’ and energetic despite sleeping less than usual and had a fierce argument with his girlfriend. On close scrutiny, it turned out that the patient's history is also positive for a single past episode of short-duration hypomania, but negative for hypomanic or manic episodes, use of antidepressants, other substance use, and medical comorbidities (Do and Mezuk 2013). However, his father suffers from severe bipolar I disorder. The patient is exhibiting a prototype hypomanic syndrome (DeFife et al. 2013), meets the DSM-5 criteria for ‘short-duration hypomanic episodes (2 to 3 days) and major depressive episodes,’ and is therefore diagnosed with ‘other specified bipolar and related disorders.’ From a clinical perspective, the current hypomanic syndrome may be the beginning of a full manic episode, particularly with the positive family history. Now consider the potential impact of three acute treatment approaches on the future diagnosis of this patient: (1) The psychiatrist prescribes a high dose of a fast-acting drug approved for the acute treatment of manic episodes, such as an atypical antipsychotic, with the possibility of further dose escalation. With this medication regimen, the hypomanic syndrome will probably decrease within a short period of time. Two days later, the patient may no longer meet the full criteria for a hypomanic episode and the diagnosis of ‘other specified bipolar and related disorders’ will remain. (2) The psychiatrist prescribes a lower dose of the same atypical antipsychotic. In this case, the patient may still meet the criteria for a hypomanic episode 2 days later and will be diagnosed with bipolar II disorder. (3) The psychiatrist decides on watchful waiting and prescribes only a mild hypnotic. The patient will develop a manic episode and will be diagnosed with bipolar I disorder.

**Table 1:** **Minimum requirements for diagnosis of selected bipolar subtypes in DSM-5**

Subtype	At least one full manic episode	At least one full major depressive episode	At least one full hypomanic episode	At least one subthreshold hypomanic episode
Bipolar I disorder	Yes	No	No	No
Bipolar II disorder	No	Yes	Yes	No
Short-duration hypomanic episodes (2 to 3 days) and major depressive episodes (other specified bipolar and related disorders)	No	Yes	No	Yes (2 episodes of short-duration hypomania)
Hypomanic episodes with insufficient symptoms and major depressive episodes (other specified bipolar and related disorders)	No	Yes	No	Yes

Similarly, long-term treatment may impact the diagnosis. Assume the patient in this example develops a hypomanic episode and is diagnosed with bipolar II disorder. Once remission is achieved, given the recent episode and family history of bipolar I disorder, prophylactic antimanic treatment is recommended in recent guidelines (Pfennig et al. 2012). The intensity of this prophylactic treatment (low to moderate dose of an atypical antipsychotic or, if further hypomanic episodes occur, a combination of this atypical antipsychotic and lithium), and the extent to which the patient adheres to this treatment, may largely influence whether this patient will develop a manic episode and subsequently be diagnosed with bipolar I disorder.

Although bipolar I and bipolar II disorders are reported to differ in clinical correlates such as age of onset or course of illness (Angst et al. 2010; Merikangas et al. 2007), these findings may be less valid in the future. With the expansion of the diagnostic criteria, a diagnosis of bipolar I disorder may be more related to treatment decisions for subthreshold bipolar II states rather than to differences in pathophysiology. In this context, the current emphasis on further delineating subtypes of bipolar disorders may be questioned. We suggest there is a need to better differentiate subthreshold hypomanic episodes from similar symptoms that occur in differential diagnoses such as normal mood swings, ADHD, narcissistic personality disorder, or borderline personality disorder (Kernberg and Yeomans 2013; Zimmerman et al. 2013). For example, in a patient presenting with a major depressive episode, probing for past ‘hyper or wired’ states with a discernible beginning and end, and including information provided by significant others, may be of help. In case of doubt, we recommend waiting until the patient has remitted and again experiences a ‘hyper or wired’ state rather than starting treatment for bipolar disorders. At this point, the patient should be reassessed by the psychiatrist and a more definite diagnosis made.

We also suggest there is an immediate need for research to more accurately capture the very essence of prototypic subthreshold hypomanic episodes. This includes prospectively and objectively measuring behavioral variables associated with subthreshold and hypomanic episodes such as communication patterns, speech, or range of motion. Projects should include the use of innovative ambulatory monitoring with wearable technologies or smartphones (Ebner-Priemer and Trull 2009; Trull and Ebner-Priemer 2013; Faurholt-Jepsen et al. 2013), in conjunction with self-ratings, and prospective clinical assessments. Better understanding of the longitudinal course of all bipolar disorder subtypes included in the DSM-5 is needed.
